# Technical validation of a new microfluidic device for enrichment of CTCs from large volumes of blood by using buffy coats to mimic diagnostic leukapheresis products

**DOI:** 10.1038/s41598-020-77227-3

**Published:** 2020-11-20

**Authors:** R. Guglielmi, Z. Lai, K. Raba, G. van Dalum, J. Wu, B. Behrens, A. A. S. Bhagat, W. T. Knoefel, R. P. L. Neves, N. H. Stoecklein

**Affiliations:** 1 Department of General, Visceral and Pediatric Surgery, University Hospital, Medical Faculty, Heinrich-Heine-University Duesseldorf, Moorenstr. 5, Bldg. 12.46, 40225 Duesseldorf, Germany; 2 Biolidics Limited, Singapore, Singapore; 3 Institute for Transplantation Diagnostics and Cell Therapeutics, University Hospital, Medical Faculty, Heinrich-Heine-University Duesseldorf, Duesseldorf, Germany; 4 Institute for Health Innovation and Technology (iHealthtech), National University of Singapore, Singapore, Singapore; 5 Department of Biomedical Engineering, National University of Singapore, Singapore, Singapore

**Keywords:** Biophysics, Cancer, Biomarkers, Oncology

## Abstract

Diagnostic leukapheresis (DLA) enables to sample larger blood volumes and increases the detection of circulating tumor cells (CTC) significantly. Nevertheless, the high excess of white blood cells (WBC) of DLA products remains a major challenge for further downstream CTC enrichment and detection. To address this problem, we tested the performance of two label-free CTC technologies for processing DLA products. For the testing purposes, we established ficollized buffy coats (BC) with a WBC composition similar to patient-derived DLA products. The mimicking-DLA samples (with up to 400 × 10^6^ WBCs) were spiked with three different tumor cell lines and processed with two versions of a spiral microfluidic chip for label-free CTC enrichment: the commercially available ClearCell FR1 biochip and a customized DLA biochip based on a similar enrichment principle, but designed for higher throughput of cells. While the samples processed with FR1 chip displayed with increasing cell load significantly higher WBC backgrounds and decreasing cell recovery, the recovery rates of the customized DLA chip were stable, even if challenged with up to 400 × 10^6^ WBCs (corresponding to around 120 mL peripheral blood or 10% of a DLA product). These results indicate that the further up-scalable DLA biochip has potential to process complete DLA products from 2.5 L of peripheral blood in an affordable way to enable high-volume CTC-based liquid biopsies.

## Introduction

Circulating tumor cells (CTCs) are cancer cells that have entered the bloodstream via passive or active mechanisms and can be traced by extremely sensitive assays in peripheral blood samples. If measured by the FDA cleared CellSearch system, detected CTCs have a strong prognostic impact in several cancer entities, including breast, colon and prostate cancer^1–3^.

The most promising aspect of CTCs is their potential use as surrogate for tissue biopsies. It is envisioned that CTCs can be analyzed to guide molecular therapies in patients with metastatic disease complementing invasive tissue biopsies, which can be uncomfortable, can have side-effects and are not always successful^4^. However, this would require to detect CTCs in almost every metastatic cancer patient, which is impossible with the available systems. For example, the CellSearch system, which is considered as current gold standard, detects relevant CTC numbers only in around 50% of metastatic cancer patients, leaving a large diagnostic gap^5^. In order to tackle this frequency problem, we introduced Diagnostic Leukapheresis (DLA) enabling the analysis of around 2.5 L of blood and thereby increasing the chance to detect CTCs^6–9^ significantly. For example, the analysis of only 5% of a generated DLA product with CellSearch resulted already in a 250% increase in CTC-detection frequency and a 30-fold escalation of CTC numbers in comparison to a normal 7.5 mL peripheral blood sample^6^. DLA is clinically safe and has been recently validated by a European multicenter study^9^. However, to unlock the full potential for CTC-based liquid biopsies, the whole DLA product or at least a large part of the DLA product would need to be processed and this is currently impeded by the high concentration of white blood cells (WBCs) and the volume of the generated blood products. Methods to process whole DLA samples (approximately 40 mL) are lacking^7^. So far, the most effective method in terms of CTC detection in DLA samples is the CellSearch system^6,9^, but considering the assay costs and the screening work-load for already one analyzed DLA sample, the CellSearch approach is not feasible for the whole product. Therefore, alternative and more economic methods for WBC depletion are urgently required. An interesting option might be CTC enrichment with spiral microfluidics. In spiral microfluidics-based approaches, the separation is label-free, which spares expensive antibodies, and it is continuous, allowing larger sample volumes to be processed. This biophysical-based CTC enrichment strategy separates smaller hematological cells from the relative larger tumor cells based on Dean forces and inertial forces generated in the curvilinear channels^10–12^. Although the performance of spiral chips for processing standard blood samples has been previously reported^10,13^, their capacity to process highly concentrated blood products such as DLA products, is unknown. Here we describe the performance of a new spiral chip that was optimized to process DLA products and compare it to a commercially available label-free spiral chip^14,15^ to process DLA samples.

## Material and methods

### Cell culture

Spiking experiments were performed using the following cell lines as models for CTCs: the human pancreatic cancer cell line Hup-T4 (DSMZ, Germany), the human breast cancer cell lines Sk-BR-3 (DSMZ) and the human prostate cancer cell line LNCaP (kindly provided by the Institute of Urology from the University Hospital and Medical Faculty of the Heinrich-Heine University Düsseldorf). All cell lines were maintained in culture under standard conditions: Hup-T4 were cultured in MEM Eagle (with EBSS, 2 mM l-Glutamine, 1 mM Sodium pyruvate, NEAA, and 1.5 g/L NaHCO3) (PAN-biotech, Germany) supplemented with 20% fetal bovine serum (FBS) (Gibco, Germany); Sk-BR-3 in McCoy’s 5A modified (with high glucose, L-Glutamine, bacto-peptone and 2.2 g/L NaHCO3) (Gibco, Germany) supplemented with 10% FBS (Gibco, Germany); LNCaP in RPMI1640 (with l-Glutamine and 2 g/L NaHCO3) (PAN-biotech, Germany) supplemented with 10% FBS (Gibco, Germany). Cells were harvested using 0.25% trypsin/EDTA (Sigma-Aldrich) for 2–5 min at 37 °C. Authenticity of the lines was verified shortly prior experiments by short tandem repeats (STRs) profiling. STR data were compared with profiles present in the DSMZ Profile Database using the respective online STR matching analysis tool (https://www.dsmz.de/fp/cgi-bin/str.html).

### Mimicking-DLA products

All experiments involving human blood donor samples were performed with the approval of the local ethics committee of the Medical Faculty of the Heinrich-Heine-University Düsseldorf, Germany (No. 4446). The experiments were performed in accordance with the relevant guidelines and regulations and ethical principles of the Declaration of Helsinki. Buffy coats (BC) were obtained from healthy blood donors, as anonymously provided by the blood donation center of the Institute for Transplantation Diagnostics and Cell Therapeutics from the University Hospital Düsseldorf (Düsseldorf, Germany), with written informed consent from each donor for the use of surplus blood products for research purposes. Data related to human samples were all analyzed anonymously. The cell content of the BC (typically around 60 mL) was analyzed within 2 h after collection using CELL-DYN Ruby hematologic analyzer (running software version 2.3ML) (Abbott, US). To prepare mimicking-DLA products (mDLA)^14^, we isolated WBCs from BC using Ficoll-Paque PLUS (d = 1.077 ± 0.001 g/mL; GE Healthcare, Sweden) or Ficoll-Paque PREMIUM (d = 1.084 ± 0.001 g/mL; GE Healthcare, Sweden) density gradient using standard protocol, washed the cells twice with PBS and analyzed again with the CELL-DYN Ruby hematologic analyzer (Supplementary figure S1B). 650 × 10^6^ WBCs were diluted to 9 mL with PBS and transferred into one Cell-free DNA BCT tube (Streck, US) for overnight fixation. After volume reduction, WBCs were resuspended to a concentration of 100 × 10^6^ WBCs/mL in ClearCell FX Resuspension Buffer or PBS containing 2% of Diluent Additive (Biolidics, Singapore), similar to the WBC concentration of DLA products (Supplementary figure S1A).

### Spike-in experiments

For optimization of CTC enrichment procedure cell culture cells were pre-labeled with Hoechst 33342 nuclear dye (ThermoFisher Scientific, USA) and one of three cytoplasmic dyes (ThermoFisher Scientific, USA) according to manufacturer protocol. Hup-T4 were pre-labelled with CellTracker Orange CMTMR Dye (CTO); Sk-BR-3 with CellTracker Green CMFDA Dye (CTG); and LNCaP with CellTracker Deep Red Dye (CTdR). These cells were fixed in Cell-free DNA BCT tubes (Streck, US) for 1 h and spiked into mDLA products using the MoFlo XDP flow sorter (Beckman Coulter, Germany) according to the following scheme: 1,000 cells of each cell line in 50 × 10^6^ WBCs equivalent to ~ 15 mL of peripheral blood (experimental condition 1); 4,000 cells of each line in 200 × 10^6^ WBCs equivalent to ~ 60 mL of peripheral blood (experimental condition 2) and 8000 cells of each line into 400 × 10^6^ WBCs equivalent to ~ 120 mL of peripheral blood (experimental condition 3) (see Supplementary Fig. S2).

### Tumor cell enrichment with CTChip FR1

Spiked-in mDLA samples were enriched using the ClearCell FX System (Biolidics Ltd, Singapore) and the respective CTChip FR1 following the instructions of the manufacturer. The CTChip FR1 consists of a spiral microfluidic channel with a rectangular cross-section (Fig. 2A) and it utilizes a Dean Flow Fractionation (DFF) principle to separate the tumor cells from the blood cells based on size differences^10,15–17^. Flowing through the spiral chip (Fig. 2A), blood cells, which are smaller than cells from solid tumors, are more heavily influenced by the secondary Dean flows (also known as a Dean vortex) which drags them to a position near the outer wall of the microfluidic channel. On the other hand, the larger tumor cells are more strongly influenced by the generated inertial lift forces and migrate (and eventually equilibrate) to a position near the inner wall. The cross-sectional dimensions of the spiral microchannels influence the forces that act on the blood cells and tumor cells. By designing appropriate spiral microchannel length and cross-sectional dimensions, the blood cells and tumor cells can be separated from one another and collected individually at the outlet position through the two separate outlets.

To test the performance of the CTChip FR1 with mDLA products, we performed three replicates in experimental condition 1, and four replicates for each cell line in experimental conditions 2 and 3 (33 experiments in total).

### Tumor cell enrichment with DLA biochip

**Figure 1:**
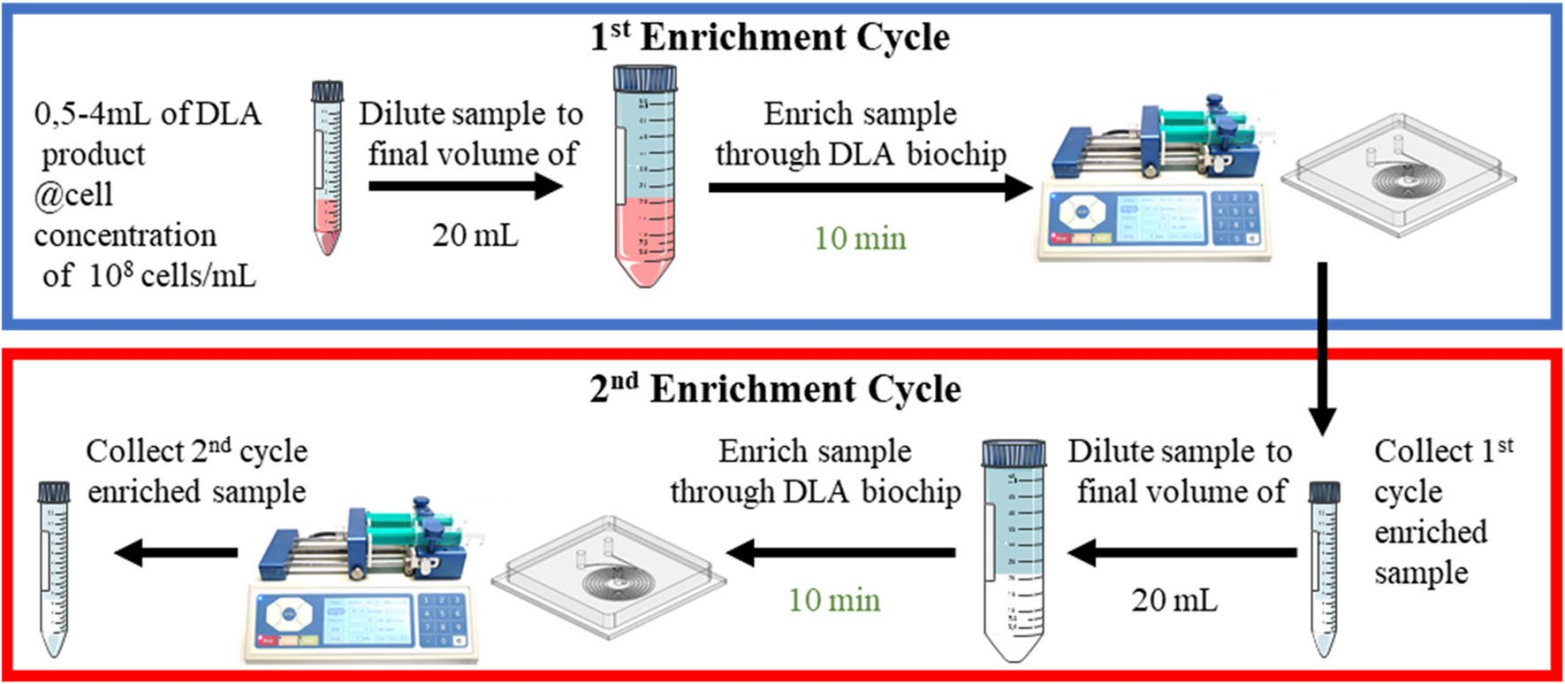
Schematic representation of DLA-enrichment workflow using the DLA biochip. 0.5, 2 and 4 mL of DLA samples with a concentration of 100 × 10^6^ WBCs/mL were diluted to 20 mL before the enrichment. The upper panel shows the first enrichment cycle through the DLA biochip which lead to a first enriched fraction (sample1), which is recovered, diluted and re-loaded through the same DLA biochip for a second enrichment cycle (lower panel).

To be processed in the DLA biochip spiked-in mDLA products were further diluted in PBS containing 2% of Diluent Additive (Biolidics Ltd, Singapore) and 1% EDTA 0,5 M up to 20 mL to favor a better cell separation. After priming the chip with PBS containing 2% of Diluent Additive, the diluted mDLA product was loaded in a 20 mL syringe (Injekt Solo, Braun) and a syringe pump channeled the fluid through the chip with a flow rate of 2.1 mL/min. Two enrichment cycles were performed through the same chip to achieve a better throughput and purity (Fig. 1). Upon the completion of the first enrichment cycle, we obtained a first waste (waste 1, W1) and a first enriched fraction (sample 1, S1). Sample S1 was re-diluted, re-loaded in the same syringe and channeled through the chip as before (Fig. 1). After this second enrichment cycle, we obtained a second waste (waste 2, W2) and a final enriched sample (sample 2, S2) (Fig. 1). The total processing time was approximately 30 min per sample.

**Figure 2:**
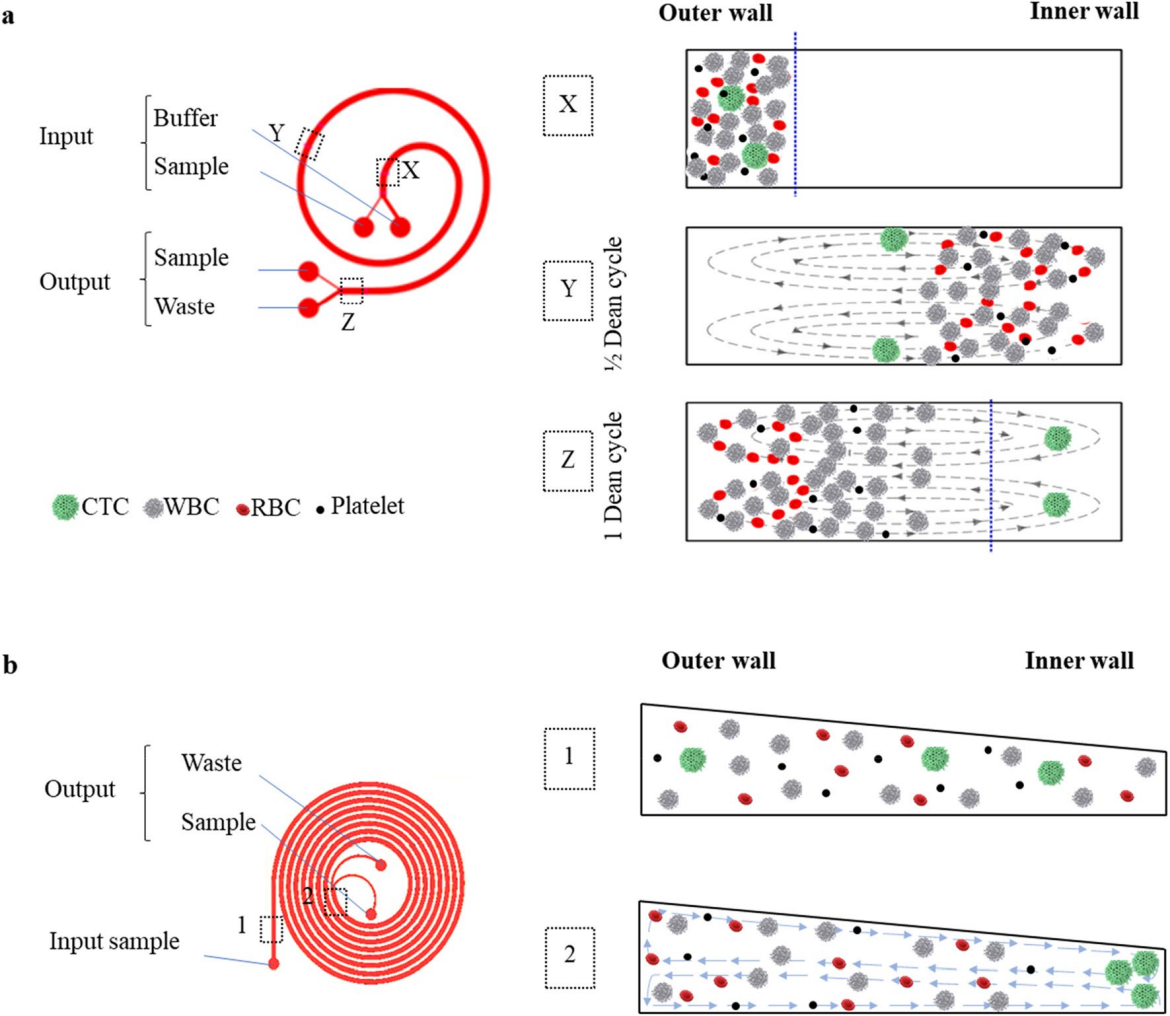
Cross-sectional views and Dean vortices generated in curvilinear microchannel for cell separation. **(a)** Adapted from^10^. Copyright 2013 by Creative Commons CC-BY-NC-ND**.** (Left panel) schematic design of CTChip FR1 showing the input slots for buffer and sample, output slots for CTCs enriched fraction and WBCs-rich waste. (Right panel) Schematic cross section showing the hematogenous cells (in white and red) and CTCs (in green) at three different positions (X, Y, Z as in the left panel). **(b)** (Left panel) schematic design of the DLA biochip showing the input slot for the pre-diluted sample and the output slots for CTCs enriched fraction and WBCs-rich waste. (Right panel) Schematic cross section showing the hematogenous cells (in grey and red) and CTCs (in green) at two different positions (1 and 2 as in the left panel).

Based on a described spiral microchannel technology with a trapezoidal cross-section^17^, a similar device was customized by Biolidics Ltd, Singapore, to process DLA products (DLA biochip) (Fig. 2B). Within this DLA biochip, separation of cells based on their sizes happens due to a Dean Vortex Trapping (DVT) process that is facilitated by the trapezoidal geometry of its cross-section. Large tumor cells in this chip behave in a similar fashion to the CTChipFR1 by migrating and eventually equilibrating in positions near the inner wall (Fig. 2). However, differing from the DFF process happening in the CTChip FR1, the secondary dean flow present in the DVT-based DLA biochip channel and which acts preferentially on the small blood cells manifests itself as a “cell trapper” entrapping the blood cells within the dean-vortexes near the outer wall^17^. This increases the probability of blood cells entering the CTC-enriched sample stream therefore resulting in poorer CTC purity. However, the inherent operating properties of this DLA biochip allows that larger sample volumes can be enriched at faster rates with potentially higher tumor cell recoveries. To increase purity of the samples two enrichment cycles through the same chip were performed.

To test the performance of the DLA biochip with mDLA products, we analyzed six replicates for each cell line for each experimental condition (54 experiments in total).

### Tumor cell detection of pre-labeled cancer cells after enrichment using flow cytometry

After enrichment of spiked mDLA samples with the CTChip FR1 or the DLA chip, the processed samples were analyzed by flow cytometry on a MoFlo XDP flow cytometer (Beckman Coulter, Germany) to enumerate the spiked cells. Intact cells were identified based on size and granularity measured as the height of Forward and Side Scatter signals (FSC-Height and SSC­Height) while single cells were discriminated from aggregates using the width of the Side Scatter signals (SSC­Width). In addition, Hup-T4 cells were defined as Hoechst^pos^/CTO^pos^; Sk-BR-3 as Hoechst^pos^/CTG^pos^; and LNCaP as Hoechst^pos^/CTdR^pos^ events (see Supplementary Fig. S3). Recovery rate was defined as the ratio between the number of detected cells and the number of initially spiked cells. In order to compare the recovery rate efficiency of the two chips, we determined a global mean tumor cell recovery rate, calculated as the mean of the recoveries of all 3 cell lines cells in all 3 experimental conditions, obtained with each technology.

### Data analysis

Statistical analyses of the data were performed in GraphPad Prism version 7.00 (GraphPad Software, San Diego, CA, USA). A non-parametric two-tailed test (Mann–Whitney U test) was used for computing statistical significances. *p* value of less than 0.05 was considered significant.

## Results

### Establishing mDLA products

**Figure 3:**
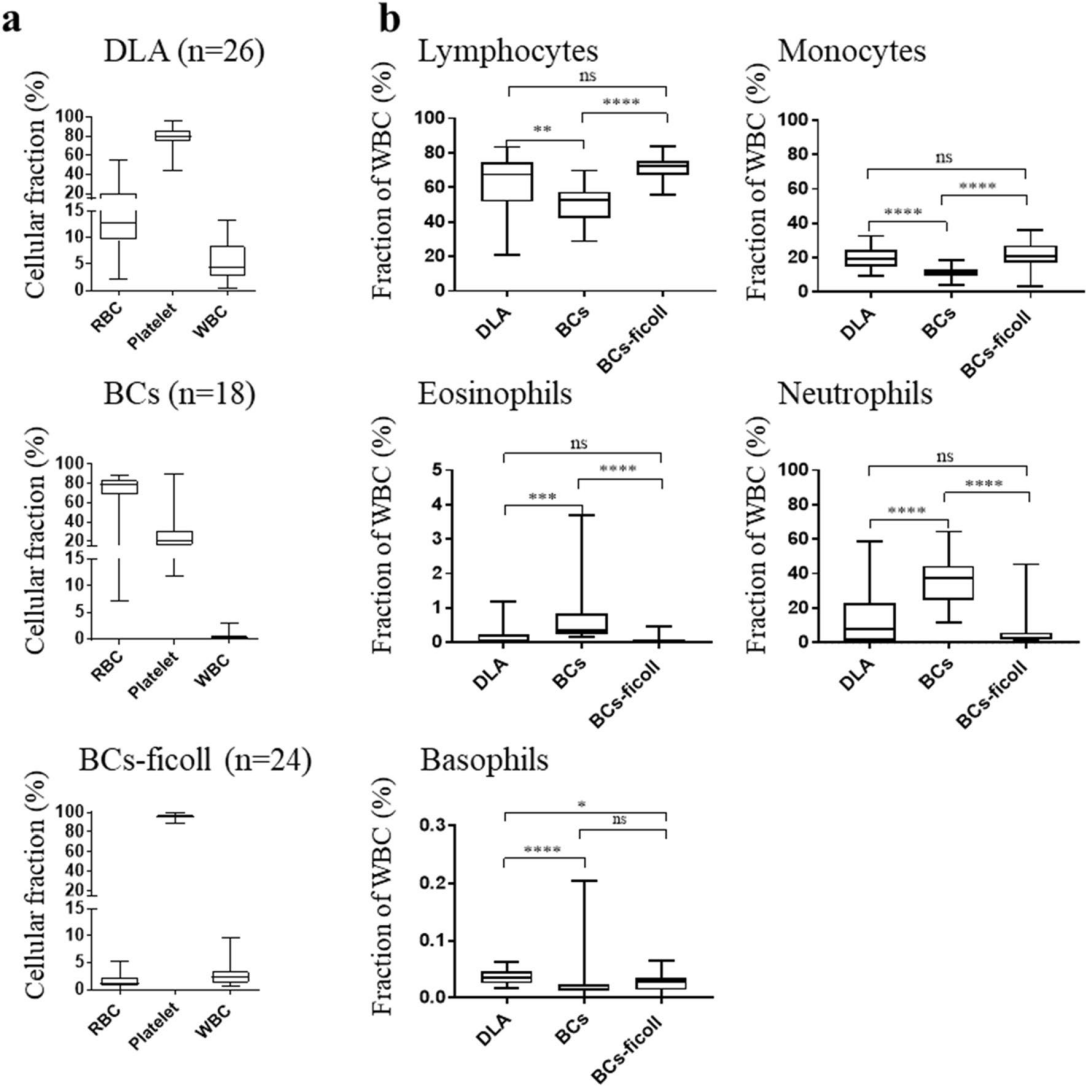
Composition of DLA samples, unprocessed and processed buffy coats. **(a)** Relative distribution of RBC, platelets and WBCs with **(b)** indication of the fraction of the different leucocyte subpopulations determined with CELL-DYN Ruby hematology analyzer and representation of the median values with interquartile range. Whiskers represent total range. The difference between the median of the percentage of five individual populations of WBCs in the three different highly concentrated blood products as in **(a)** was compared. Statistical analysis (Mann–Whitney *U* test*):* *P < .05; **P < .01; ***P < .001; ****P < .0001.

Since the availability of DLA products for testing new applications and protocols is very limited, we sought alternative, more easily available blood products for testing purposes. Obvious candidates were BC prepared by the centrifugation of anti-coagulated whole blood and consisting of a concentrated suspension of WBCs and platelets. BCs are available in larger quantities (50–100 mL) for research purposes from blood banks or commercial providers. In order to test whether BCs can be used as surrogates for DLA products, we compared their blood cell profiles. BC collected from healthy donors (n = 18) had on average higher red blood cell (RBC) fraction (p < 0.0001), lower platelet fraction (p < 0.0001), and lower WBC fraction (p < 0.0001) compared to patient-derived DLA products (n = 26) (Fig. 3A). Because nucleated cells are the most relevant and challenging blood cells for CTC enrichment (Supplementary Fig. S1), we focused on the different WBC populations. We observed a significant difference in the prevalence (unpaired Mann–Whitney U tests) of each WBCs population between DLA products and BCs (Fig. 3B). In order to obtain better DLA surrogates, we tested processing BC with density gradient centrifugation employing the standard Ficoll-Paque PLUS (d = 1.077 g/mL) which would preferentially enrich the mononuclear cells with lower density (1.067–1.077 g/mL) and the Ficoll-Paque PLUS 1.084 g/mL with a density outside the density window targeted by DLA procedure (1.055–1.08 g/mL) and less sharp for granulocytes with higher density (> 1.080 g/mL)^18^. Surprisingly, no significant difference between WBC composition after centrifugation with the two different media could be observed and both media resulted in a WBC composition similar to DLA products (see Supplementary Fig. S5), indicating that both could be used alike to produce suitable mDLA products. We considered the fact that the basophil counts were still lower compared to DLA products (Fig. 3B) as negligible due to the extremely low prevalence of this population.

### Size determination of the different cancer cell lines

Since the cell size influences the cell enrichment efficiency on spiral microfluidics chips, we determined the diameter of the three cell line cells tested here. We measured a mean diameter of 13.7 ± 1.4 µm (range 10.5 to 15.9 µm) for Sk-BR-3, 15.1 ± 2.4 µm (range 11.8 to 20.5) for Hup-T4, and 15.9 ± 3.4 µm (range 12.3 to 26.4), for LNCaP (see Supplementary Fig. S6). Although minor differences in sizes were observed (see Supplementary Fig. S6), we expected a similar performance for all three cell lines. In contrast, the diameter for the WBCs (8.5 ± 0.9 µm, range 6.4 to 10 µm) was significantly smaller than the diameter of the cell line cells (p < 0.0001).

### Recovery of pre-labeled tumor cells spiked in mDLA samples enriched using the ClearCell FX system and the DLA biochip

**Figure 4:**
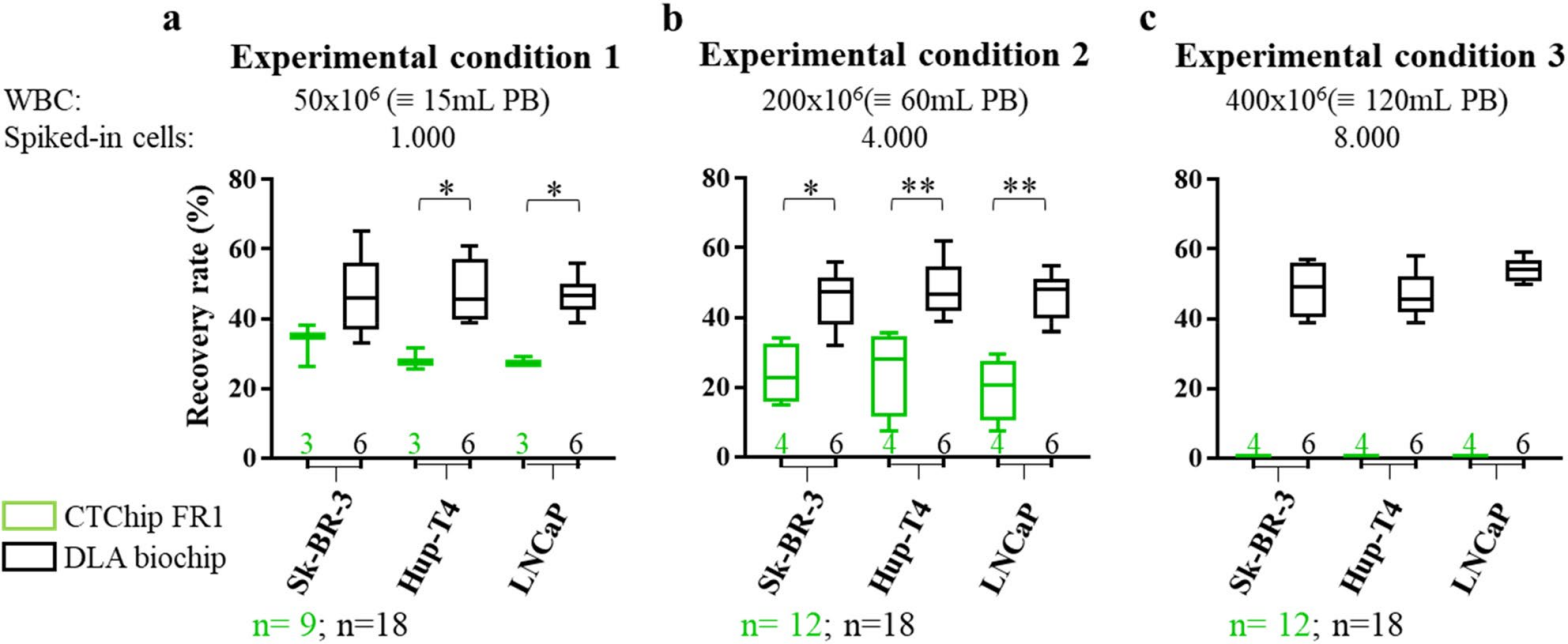
Spiked-in cells recovery rates after enrichment with the CTChipFR1 and DLA biochip. **(a)** 1000, **(b)** 4000 and **(c)** 8000 cancer cells from each of three differentially pre-labelled cell lines were spiked with flow cytometry respectively into **(a)** 50 × 10^6^, **(b)** 200 × 10^6^ and **(c)** 400 × 10^6^ WBCs from mimicking-DLA products. Enriched cells were enumerated again by flow cytometry and compared with the number of initially spiked cells to determine recovery rates. The difference between median recovery rates in percentage were compared after enrichment with the ClearCellFX System (green boxes) and DLA biochip (black boxes). Whiskers represent total range. Statistical analysis (Mann–Whitney *U* test): *P < 0.05; **P < 0.01.

Next, we used the mDLA samples to challenge the spiral chip technology. As the commercially available ClearCell FX platform with the CTChip FR1 had been designed for peripheral blood samples, we initially tested recovery on this system using samples with 50 × 10^6^ WBCs (equivalent to ~ 15 mL PB, experimental condition 1, n = 3 for each cell line) (Fig. 4A). The mean tumor cell recovery rate from mDLAs containing 50 × 10^6^ WBCs for all three cell lines was 30% (n = 9). Next, we tested the device with mDLA samples containing 200 × 10^6^ WBCs (equivivalent to ~ 60 mL PB, experimental condition 2, n = 4 for each cell line) (Fig. 4B) and 400 × 10^6^ WBCs (equivivalent to ~ 120 mL PB, experimental condition 3, n = 4 for each cell line) (Fig. 4C). With the higher WBC number in experimental condition 2, the global mean recovery rate dropped to 23% (n = 12) (Fig. 4B) and in all experiments using the experimental condition 3 the output straw of the device clogged and did not allow sample enrichment (Fig. 4C). The global mean tumor cell recovery rate with the CTChip FR1 in experimental conditions 1 and 2 was 25% (n = 21).

We next tested the DLA biochip for processing similar samples. For a direct comparison with the CTChip FR1 chip, the mDLA products were prepared according to the same experimental workflow as used for the CTChip FR1 and containing 50 × 10^6^, 200 × 10^6^ and 400 × 10^6^ WBCs (n = 6 for each cell line in each experimental condition) (Fig. 4). All enrichment cycles were performed without any visible clogging or other problems concerning the flow through the microfluidic chip. Despite a higher recovery rate for LNCaP in mDLA samples with 400 × 10^6^ WBCs, no significant recovery differences between the different cell lines were noted. The mean tumor cell recovery rate from mDLAs containing 50 × 10^6^ WBCs (equivivalent to ~ 15 mL PB) was 47% (n = 18), containing 200 × 10^6^ WBCs (equivalent to ~ 60 mL PB) was 47% (n = 18), and containing 400 × 10^6^ WBCs (equivalent to ~ 120 mL PB) was 50% (n = 18), and those recoveries were not statistically different across the three experimental conditions. The global mean tumor cell recovery rate for the DLA biochip was 48% (n = 54) and significantly higher than the recovery calculated for the FR1 chip.

### WBC contamination after DLA biochip enrichment

**Figure 5:**
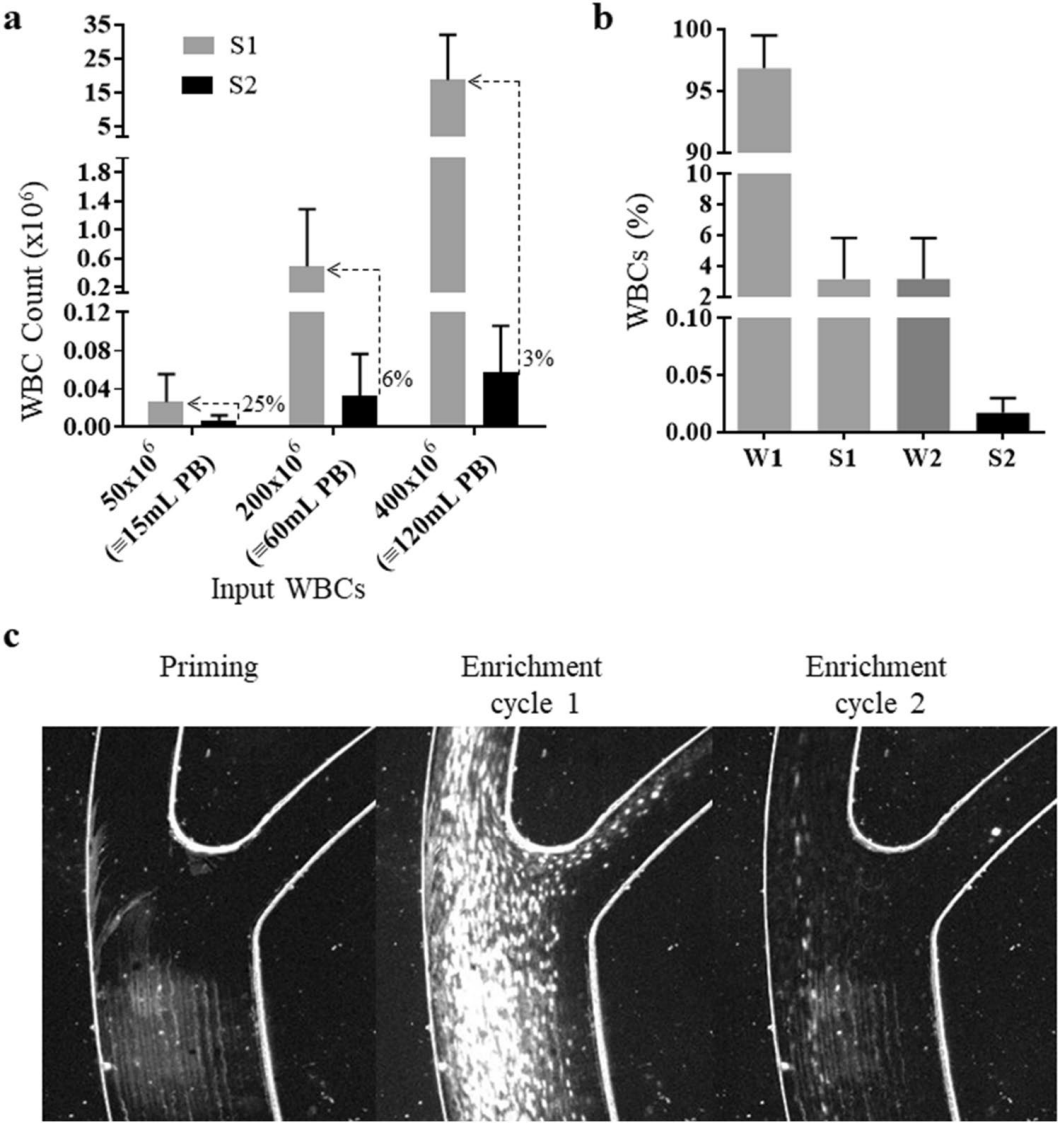
Evaluation of WBC contamination after one and two cycles of enrichment with the DLA biochip. mDLA products containing 50 × 10^6^, 200 × 10^6^ and 400 × 10^6^ WBCs were enriched with the DLA biochip and the **(a)** Mean number of WBCs in the enriched fraction after one (S1, analyzed with CELL-DYN Ruby hematology analyzer), and two enrichment cycles (S2, analyzed with flowcytometry). Lines represent standard deviation. **(b)** Mean percentages with standard deviation of WBCs in mDLA products containing 400 × 10^6^ WBCs and enriched with the DLA biochip were determined in the S2, in the waste of the first enrichment cycle (W1) and in the waste of the second enrichment cycle (W2). **(c)** Representative snapshot of the WBCs flow during the first and the second enrichment cycle, tracked using the Photron Fastcam SA3 (MEC, Indiana) connected to the Olympus IX71 inverted microscope (Olympus, US).

CTC-screening or other subsequent applications (e.g. molecular analyses) might be further challenging after enrichment in case of high WBC carryover. To get information on the level of WBC carryover and on the importance of the second enrichment cycle, we determined the WBC content remaining in the sample after the first (S1) and second (S2) round of DLA biochip enrichment (Fig. 5A). As expected (Fig. 5C), the number of co-enriched WBCs correlated positively with the number of input cells and decreased after performing the second round of enrichment. We also determined the distribution of the input cells through the different cellular fractions generated during DLA biochip enrichment of mDLA samples (waste of cycle 1 (W1), and samples after enrichment cycles 1 and 2 (S1 and S2) (Fig. 5B). We determined that 96.84% ± 2.66% (s.d.) of the initial WBCs were present on the waste of the first enrichment cycle (W1) and an additional 3.14% ± 2.65% (s.d.) were present on the waste of the second enrichment cycle (W2) (Fig. 5B). In the final enriched sample obtained after the two cycles (S2) we could find 0.017% ± 0.013% (s.d.) (which corresponded to an average of 60.000 WBCs).

## Discussion

DLA is a promising^6^ and clinically safe^8^ approach to screen liters of blood for CTCs. While its dramatic effect on CTC detection frequency and yield has been already validated^9^, investigating the DLA products for CTCs remains challenging. DLA is a pre-enrichment approach that requires additional enrichment and detection steps, for example, like those done with the CellSearch system. But although this method has been so far the most effective to detect CTCs in DLA products, it is limited to 200 × 10^6^ WBCs per run (equivalent to ~ 60 mL of PB and around 5% of a typical 40 mL DLA product^6,9^). Therefore, more scalable and economic approaches are needed to unlock the full potential of DLA. In this context, microfluidic-^19,20^ or filtration-based^21^ label-free CTC enrichment methods appear interesting (despite used so far only with 100 × 10^6^ cells), because they might allow a more continuous sample processing with the possibility to analyze higher sample volumes in a cost-effective manner (e.g. by avoiding expensive conjugated antibody cocktails for positive enrichment). Particularly promising are hydrodynamic-based spiral microfluidic devices, that take advantage of Dean and inertial forces to continuously separate the cells^22^. For our present work, we tested a novel inertial microfluidic based spiral biochip that was capable to process up to 4 mL DLA samples with high WBC content (400 × 10^6^ WBCs, equivalent to ~ 120 mL PB). Although this represents only 10% of the DLA volume, the approach is easily scalable by repeating the process on the chip with further portions of the same DLA product and/or by running several chips in parallel. Importantly, our data indicated a good recovery rate for all three different cell lines independent from the WBC input. This can be explained by intrinsic properties of the DLA biochip with its trapezoidal cross-section, whose design is based on similar existing devices^17,23^, but optimized to process WBC-rich DLA products allowing maximal WBC depletion and CTC retention. Indeed, it was already demonstrated that a trapezoidal channel cross-section can increase the resolution of size-based separation of WBCs from the more abundant RBCs. The trapezoidal geometry alters the shape of velocity field and contributes to a better positioning of smaller cells near the outer wall, far enough to not disturbing the position of larger cells near the inner wall. This improved separation with minimized interaction between particles with different sizes allows devices with trapezoidal geometry to process higher number of background^17,24^. The chip design is simple and easy to operate despite requiring two-cycle enrichment for DLA samples. Importantly, the DLA biochip did not clog as previously reported for filtration devices^25^, for deterministic lateral displacement (DLD) methods^26^, and as we could observe by processing mDLAs with the highest WBCs counts using the CTChip FR1 (Fig. 4C). It is conceivable that the larger dimensions of the DLA biochip channels, when compared to the standard^27^, might help to prevent clogging. Moreover, the trapezoidal cross-section of the spiral chip, coupled with a larger number of turns, allowed to operate the chip in a sheath-less configuration unlike the CTChip FR1. We believe that all of these important characteristics of the technology contributed to the successful processing and tumor cell enrichment of samples with high WBC concentrations.

Although we are aware of the fact that tumor cell lines cannot properly reflect the heterogeneous patient´s CTCs, they can help to determine the technical performance of a new enrichment method in a standardized fashion. Therefore, we tested the spiral chips with cell lines frequently used in CTC assays and covering the spectrum of three major cancer entities. Since it is difficult to use precious DLA samples for establishing new methods or technical validations, we evaluated if BC after density gradient centrifugations might represent a good surrogate of patient-derived DLA based on its hematological composition. Our data (Fig. 3) provides evidence that the WBC composition of the mDLA is similar to the patient-derived DLA products highly commending them for standardized testing purposes. Our data revealed the superiority of the DLA biochip over the ClearCell FX System for further CTC-enrichment of DLA products, even if challenged with up to 400 × 10^6^ WBCs per run (equivalent to ~ 120 mL PB). The suitability of the CTChip FR1 for our purposes was already questioned when comparing the tumor cell recoveries of mDLAs with a WBC content of 50 × 10^6^ WBCs. Based on own previous experiments done with 7.5 mL blood samples, we expected a tumor cell recovery in average of 60% (data not shown) or > 85% based on data from the literature^10^. Notably, the processing of mDLAs containing just 50 × 10^6^ WBCs (equivalent to ~ 15 mL) already resulted in a mean tumor cell recovery of just 30% (Fig. 4A), which might be in part explained by the different rheological properties of DLA products compared to peripheral blood, but also reveal the limitations of this system to operate outside its intended use.

A further relevant aspect that determines the performance of a CTC enrichment method is the WBCs contamination in the enriched fraction. In fact, a high WBC carryover might be not compatible with CTC detection and isolation methods or may even interfere with CTC-specific gene expression profiling^28^. For the case of DLA products with their excessive WBC counts, this issue becomes more significant. In this aspect, the DLA biochip revealed a high WBC depletion of 3.9 ± 0.3 logs after the two cycles corresponding to a depletion of 99.98% of WBCs. Importantly, the second enrichment cycle alone was responsible for removing around 3% of WBCs (equivalent to approximately 12 × 10^6^ WBCs on experimental condition 3).

In summary, our results suggest the newly designed spiral DLA biochip as a promising tool to enrich CTCs from DLA products with a relatively low carryover of WBCs. The scalability of the method further suggests that it could potentially allow the processing of complete DLA products.

## Supplementary information

Supplementary information.
