# Supplementary material for: Technical validation of a new microfluidic device for enrichment of CTCs from large volumes of blood by using buffy coats to mimic diagnostic leukapheresis products
Source: Sci Rep. 2020 Nov 20;10:20312. doi: 10.1038/s41598-020-77227-3 (PMC7680114; doi:10.1038/s41598-020-77227-3)
Supplement: Supplementary file 1 — Supplementary information. [file 41598_2020_77227_MOESM1_ESM.pdf]

TECHNICAL VALIDATION OF A NEW MICROFLUIDIC DEVICE FOR ENRICHMENT OF CTCs FROM LARGE VOLUMES OF BLOOD BY USING BUFFY COATS TO MIMIC DIAGNOSTIC LEUKAPHERESIS PRODUCTS.

AUTHORS

R. Guglielmi[1], Z. Lai[2], K. Raba[3], G. van Dalum[1], J. Wu[1], B. Behrens[1], A. A. S. Bhagat[4,5], W.T. Knoefel[1], R. P. L. Neves[1,6] and N. H. Stoecklein[1,6,*]

AFFILIATIONS

[1]Department of General, Visceral and Pediatric Surgery, University Hospital and Medical Faculty of the Heinrich-Heine-University Duesseldorf, Duesseldorf, Germany.

[2]Biolidics Limited, Singapore.

[3]Institute for Transplantation Diagnostics and Cell Therapeutics, University Hospital and Medical Faculty of the Heinrich-Heine-University Duesseldorf, Duesseldorf, Germany.

[4]Institute for Health Innovation and Technology (iHealthtech), National University of Singapore, Singapore.

[5]Department of Biomedical Engineering, National University of Singapore, Singapore.

[6]The authors contributed equally to this manuscript

[*]Corresponding author

CORRESPONDING AUTHOR

Nikolas H. Stoecklein
University Hospital and Medical Faculty of the Heinrich-Heine-University Duesseldorf, Moorenstr. 5, Bldg. 12.46, 40225 Duesseldorf, Germany
Tel: + 49 211 81 16399Fax: + 49 211 81 04109
E-mail: nikolas.stoecklein@hhu.de

SUPPLEMENTARY FIGURE 1

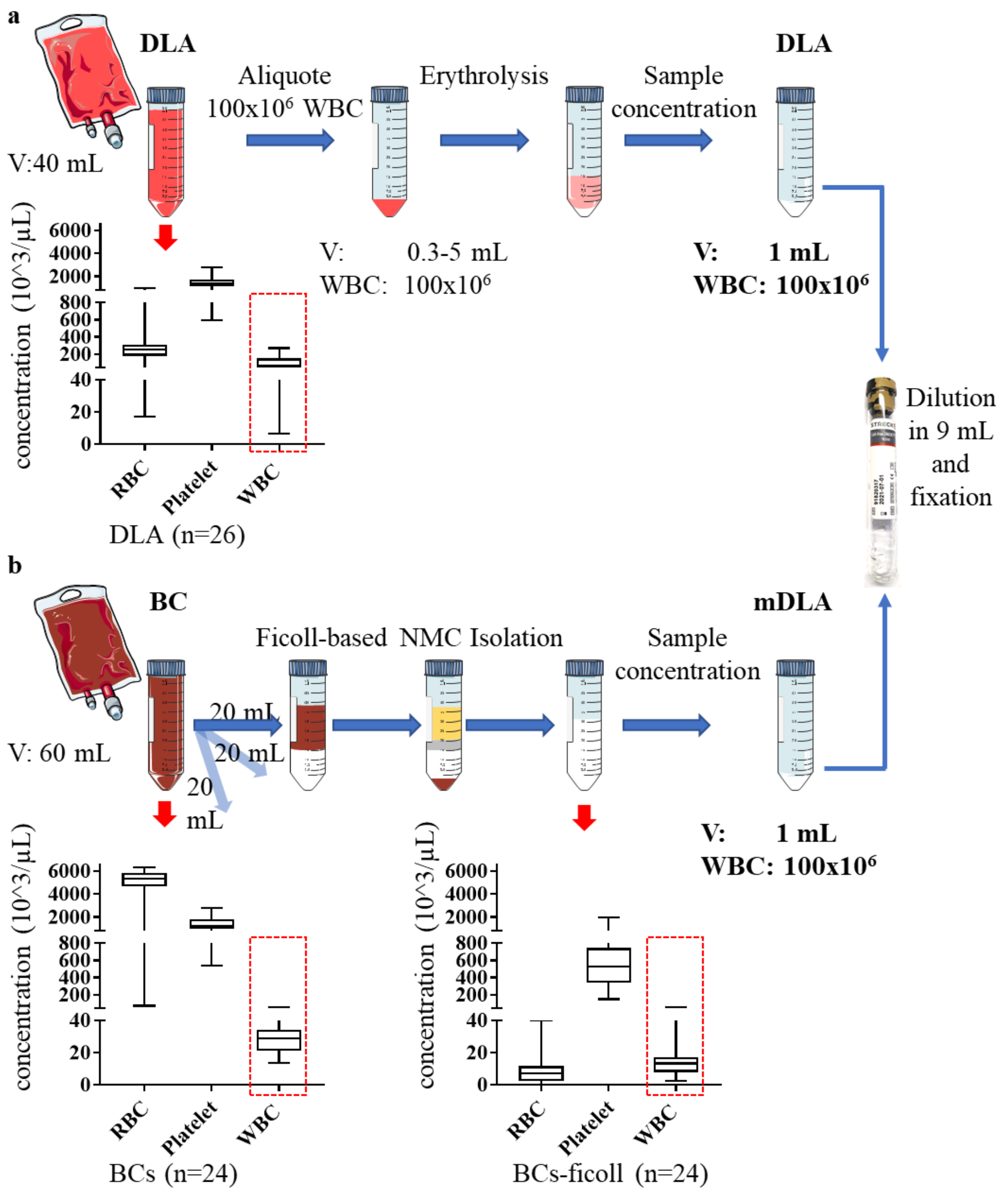

Supplementary Fig. S1 - Optimized protocols to obtain DLA and mDLA with a WBC concentration of 100x10^6 WBC/mL available for enrichment with ClearCell FR1 biochip and DLA biochip. (a) The cell content of the DLA products (typically around 50 mL) was analyzed after collection using CELL-DYN Ruby hematologic analyzer. To prepare DLA products for the enrichment, aliquotes with 100x10^6 WBC are prepared and the sample is concentrated to 100x10^6 WBC/mL after RBC lysis. **(b)** The cell content of the BC (typically

around 60 mL) was analyzed using CELL-DYN Ruby hematologic analyzer. To prepare mimicking-DLA products (mDLA) for the enrichment, the WBC are isolated from BC using Ficoll-Paque (GE Healthcare, Sweden) or Ficoll-Paque PREMIUM (GE Healthcare, Sweden) density gradient, washed twice with PBS and analyzed again with the CELL-DYN Ruby hematologic analyzer. The sample is concentrated to 100×10^6 WBC/mL in ClearCell FX Resuspension Buffer or PBS containing 2% of Diluent Additive (Biolidics, Singapore).

The products enriched in WBCs are ready for further dilution and overnight fixation in Cell-free DNA BCT tube (Streck, US). The falcon tubes, the DLA and BC bags used in this figure were extracted from "Medical Equipment" attributed to Servier Medical Art. These were used and adapted under a Creative Commons Attribution 3.0 Unported License.

SUPPLEMENTARY FIGURE 2

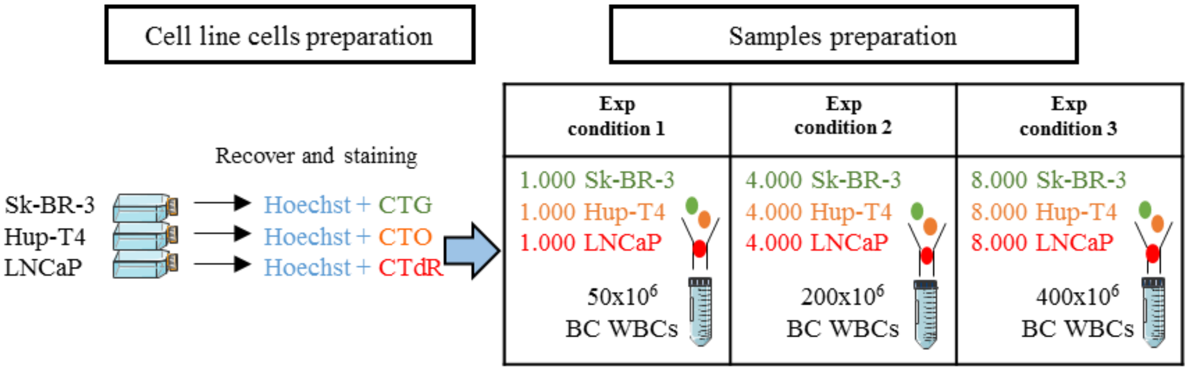

Supplementary Fig. S2 - Experimental setup for spike-in experiments. Cell culture cells were harvested and pre-labeled with a nuclear staining (Hoechst 33342) and one of three different cytoplasmic dyes: CellTracker Orange CMTMR Dye (CTO); CellTracker Green CMFDA Dye (CTG); or CellTracker Deep Red Dye (CTdR). Using these pre-labeld cells, three different sample types (experimental conditions) were prepared. 1.000, 4.000 and 8.000 cancer cells from each differentially pre-labelled cell line were spiked with flow cytometry respectively into $50x10^6$, $200x10^6$ and $400x10^6$ WBCs from mimicked DLA products. The falcon tubes and the cell culture flasks in this figure were extracted from "Medical Equipment" attributed to Servier Medical Art. These were used and adapted under a Creative Commons Attribution 3.0 Unported License.

SUPPLEMENTARY FIGURE 3

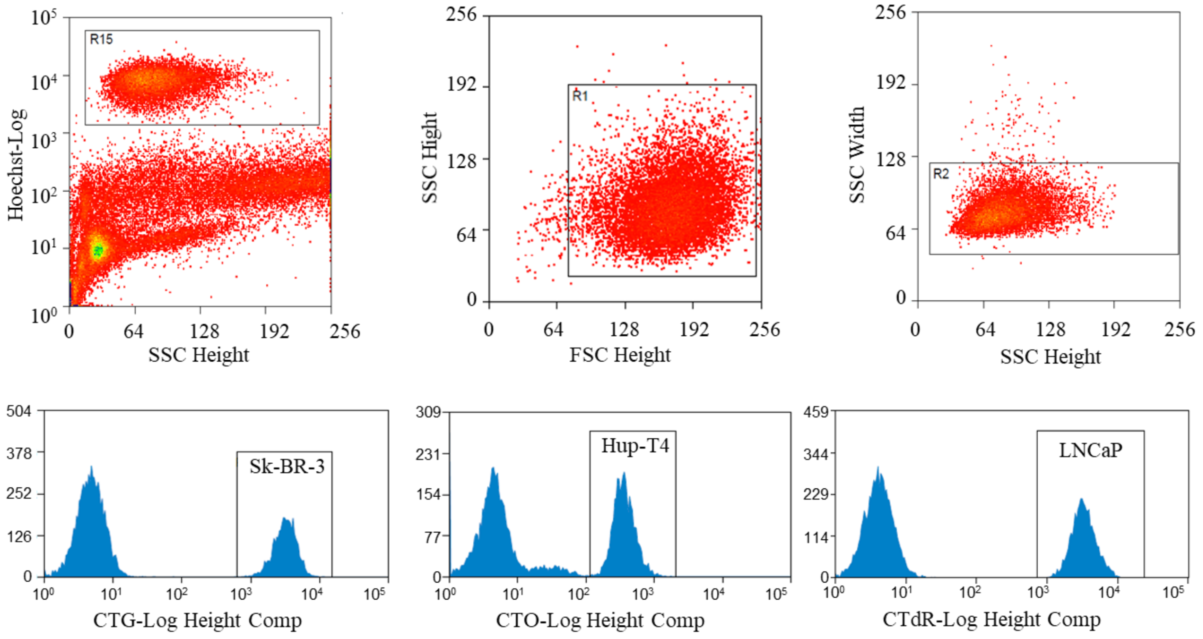

Supplementary Fig. S3 - Detection of pre-labeled tumor cells using flow cytometry after enrichment. In this representative experiment, 8.000 cancer cells from each of three differentially pre-labeled cell lines were spiked into a mimicked DLA product with 400x10^{6} WBCs (4mL sample). The sample was processed with the DLA biochip and the whole output was analyzed by flow cytometry using the MoFlo XDP flow sorter (Beckman Coulter, Germany) to detect contemporarily the three differentially pre-labeled tumor cells. The dotplots show the sequence of gates used to identify nucleated (left plot), intact (central plot), and single (right plot) cells. The cells identified with these three gates were further analysed for their fluorescence for CellTracker Orange CMTMR Dye (CTO); CellTracker Green CMFDA Dye (CTG); or CellTracker Deep Red Dye (CTdR) and the number of events positive for the different fluorophores was determined using the gates shown in the histograms.

SUPPLEMENTARY FIGURE 4

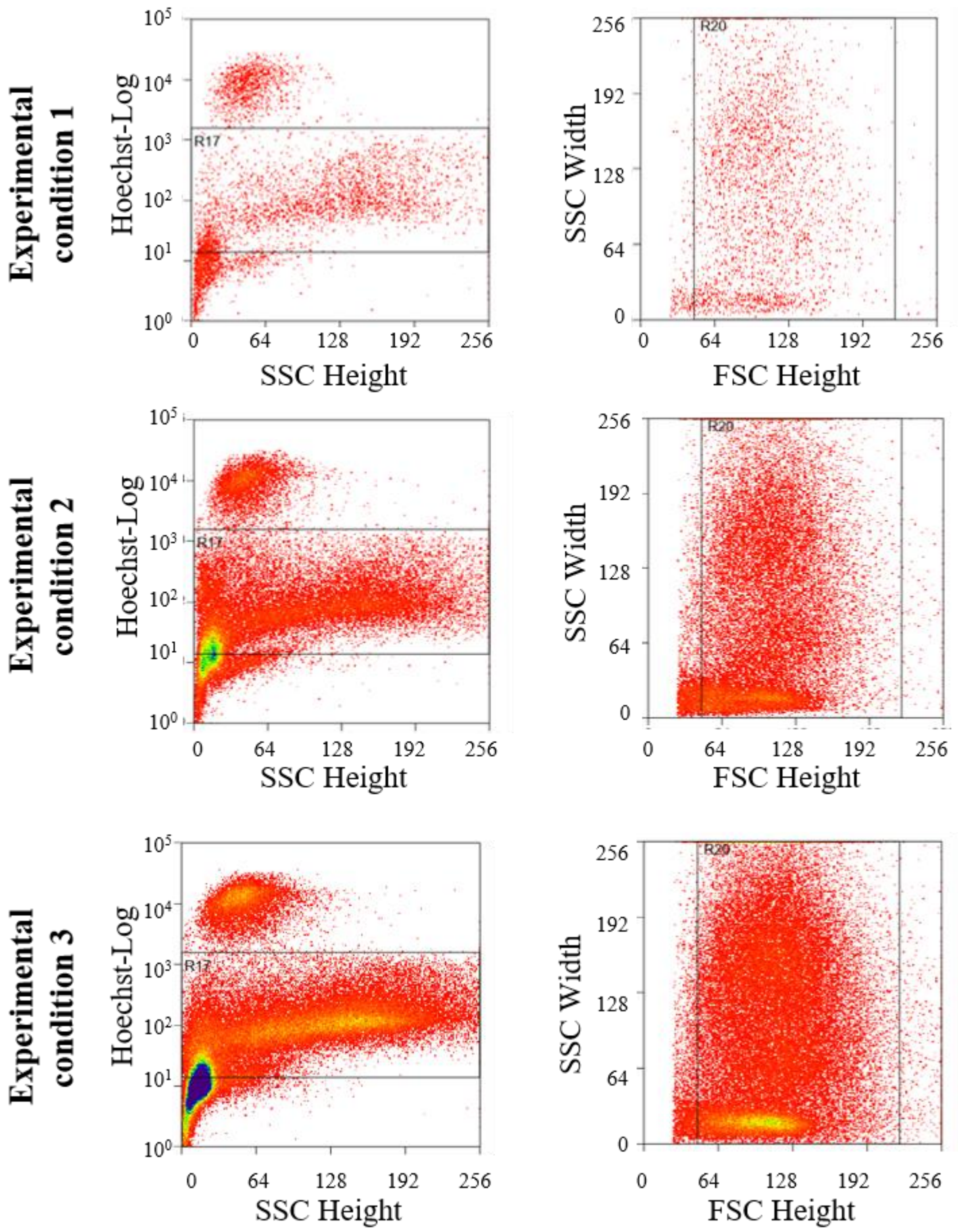

Supplementary Figure S4. Detection of the contaminating cells using flow cytometry after two enrichment cycles. In these representative experiments, 8.000, 4.000 and 1.000 cancer cells from each of three differentially pre-labeled and Hoechst pre-stained cell lines were spiked respectively into a mimicked DLA product with $50x10^6$ (experimental condition 1), $200x10^6$ (experimental condition 2) and $400x10^6$ (experimental condition 3) unstained WBCs. The sample was processed through the DLA biochip (2 rounds) and the whole output (sample S2) was analyzed by flow cytometry using the MoFlo XDP flow sorter (Beckman Coulter, Germany). The plots represent the sequence of gates used to identify Hoechst negative (left plot) and intact (right plot) cells.

SUPPLEMENTARY FIGURE 5

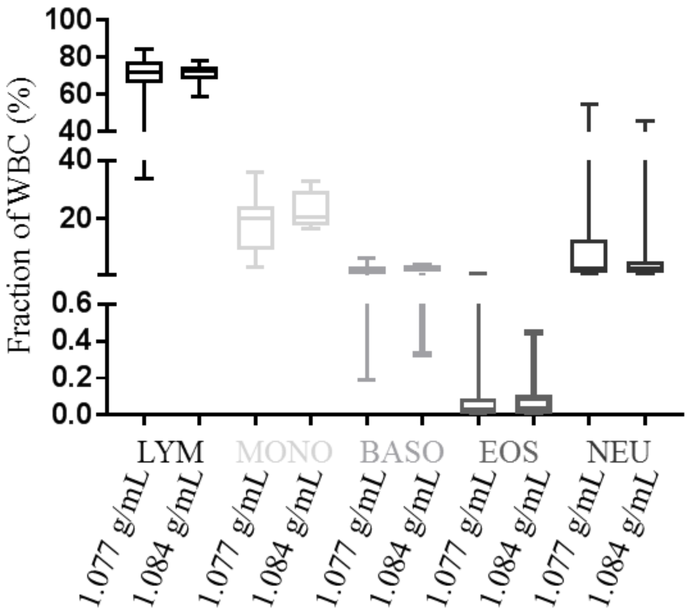

Supplementary Fig. S5 - Comparison of leucocytes composition of BC after density gradient centrifugation performed with two different Ficoll media. Products obtained after gradient centrifugation with Ficoll-Paque 1.077 g/mL (n=13) or 1.084 g/mL (n=13) were analyzed by CELL-DYN hematology analyzer to determine the content of each WBC subpopulations. The results shown are here expressed as the percentage of the complete WBC population. Whiskers represent total range and lines represent median values. The fractions of each WBC subpopulation were not significantly different (Mann-Whitney *U* test) after centrifugation with the two media.

SUPPLEMENTARY FIGURE 6

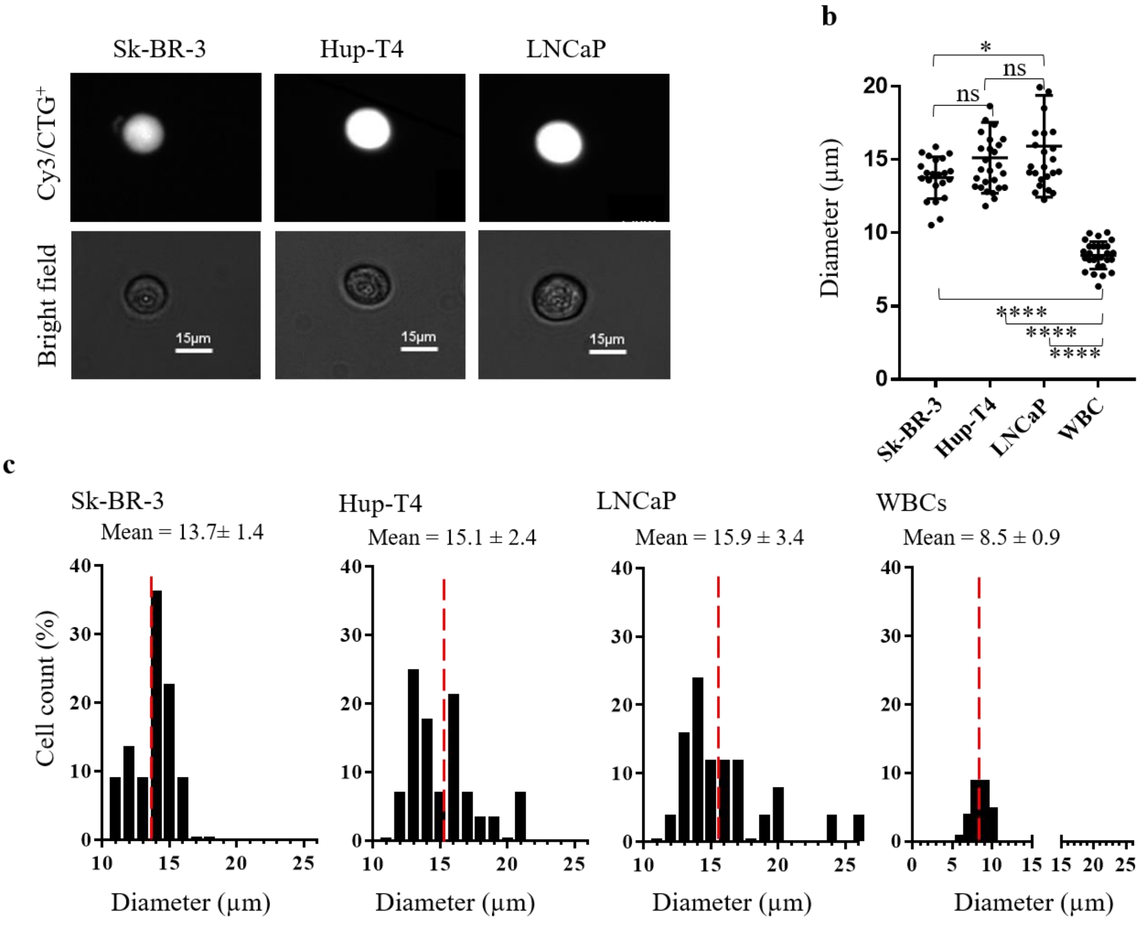

Supplementary Fig. S6 - Cell size of the three tumor cell lines used and of WBCs from mDLA. Sk-BR-3, LNCaP and Hup-T4 cells harvested from culture and WBCs contained in mDLA, for the specific purpose of size assessment, were all similarly pre-labeled with CellTracker Green CMFDA Dye (CTG) (ThermoFisher Scientific, USA). This dye allowed sharp cellular contours for more accurate determination of cellular dimensions. After staining, a small number of cells was transferred into one 14mm-field of a 3-field adhesive slide for microscopy (Erie Scientific LLC, NH, USA) to favor a good cell dispersion. Microscopy slide-fields were automatically scanned in an Eclipse E400 epi-fluorescence microscope (Nikon, Japan). Subsequently, the obtained images were analyzed with ImageJ software to determine cell diameters in pixels, which were afterward converted in µm. Among cells with intact morphology, we randomly selected n=22 Sk-BR-3 cells, n=28 Hup-T4 cells, n=25 LNCaP cells and n=28 WBCs. **(a)** Representative microscopy imagines of pre-labeled Sk-BR-3, Hup-T4 and LNCaP obtained with Cy3 fluorescence filter and bright field. **(b)** Diameter determined for individual cells of the three different cell lines and WBCs. Horizontal lines represent mean and vertical lines represent standard deviation. Statistically significant differences (Mann-Whitney *U* test, P = 0.0194) could only be observed between Sk-BR-3 and LNCaP cells. **(c)** Frequency distribution of cell diameter with the indication of the mean value (± standard deviation) for each tumor cell line and WBC.
